# The microbiome of the ice-capped Cayambe Volcanic Complex in Ecuador

**DOI:** 10.3389/fmicb.2023.1154815

**Published:** 2023-05-05

**Authors:** Magdalena Díaz, Pablo Monfort-Lanzas, Cristian Quiroz-Moreno, Erika Rivadeneira, Pablo Castillejo, Vicente Arnau, Wladimiro Díaz, Spiros N. Agathos, Félix J. Sangari, Pablo Jarrín-V, C. Alfonso Molina

**Affiliations:** ^1^Departamento de Biología Molecular, Universidad de Cantabria, Santander, Spain; ^2^Instituto de Investigación en Zoonosis (CIZ), Universidad Central del Ecuador, Quito, Ecuador; ^3^Facultad de Ingeniería Química, Universidad Central del Ecuador, Quito, Ecuador; ^4^Institute of Integrative Systems Biology (I^2^SysBio), University of Valencia and Consejo Superior de Investigaciones Científicas (CSIC), Valencia, Spain; ^5^Department of Horticulture and Crop Science, Ohio State University, Columbus, OH, United States; ^6^Grupo de Investigación en Biodiversidad, Medio Ambiente y Salud (BIOMAS), Universidad de las Américas, Quito, Ecuador; ^7^Facultad de Ingeniería y Ciencias Aplicadas, Universidad Internacional SEK, Quito, Ecuador; ^8^Earth and Life Institute (ELI), Université Catholique de Louvain, Louvain-la-Neuve, Belgium; ^9^Instituto de Biomedicina y Biotecnología de Cantabria (IBBTEC), CSIC – Universidad de Cantabria, Santander, Spain; ^10^Dirección de Innovación, Instituto Nacional de Biodiversidad INABIO, Quito, Ecuador; ^11^Facultad de Medicina Veterinaria y Zootecnia, Universidad Central del Ecuador, Quito, Ecuador

**Keywords:** volcano, bacterial community, Andean glacier, elevational gradient, diversity

## Abstract

A major challenge in microbial ecology is to understand the principles and processes by which microbes associate and interact in community assemblages. Microbial communities in mountain glaciers are unique as first colonizers and nutrient enrichment drivers for downstream ecosystems. However, mountain glaciers have been distinctively sensitive to climate perturbations and have suffered a severe retreat over the past 40  years, compelling us to understand glacier ecosystems before their disappearance. This is the first study in an Andean glacier in Ecuador offering insights into the relationship of physicochemical variables and altitude on the diversity and structure of bacterial communities. Our study covered extreme Andean altitudes at the Cayambe Volcanic Complex, from 4,783 to 5,583 masl. Glacier soil and ice samples were used as the source for 16S rRNA gene amplicon libraries. We found (1) effects of altitude on diversity and community structure, (2) the presence of few significantly correlated nutrients to community structure, (3) sharp differences between glacier soil and glacier ice in diversity and community structure, where, as quantified by the Shannon γ-diversity distribution, the meta-community in glacier soil showed more diversity than in glacier ice; this pattern was related to the higher variability of the physicochemical distribution of variables in the former substrate, and (4) significantly abundant genera associated with either high or low altitudes that could serve as biomarkers for studies on climate change. Our results provide the first assessment of these unexplored communities, before their potential disappearance due to glacier retreat and climate change.

## 1. Introduction

For over two decades, climate change has been considered a significant threat to vulnerable ecosystems, such as glaciers and ice-capped volcanoes, which are affected by sharp changes in temperature (Oerlemans, 1994). Global melting and glacier retreat is one main effect of climate change (Shi and Liu, 2000; Raper and Braithwaite, 2006; Sorg et al., 2015). The retreat of tropical Andean glaciers is considered a climate change indicator, particularly as glaciers are sensitive to climate perturbations (Rabatel et al., 2013, 2018). A consistent retreat over the past 40 years has been evident at various Andean glaciers (Małecki et al., 2018). It is therefore important to understand glacier ecosystems in the Andes before their possible disappearance (Stibal et al., 2020).

**Figure 1:**
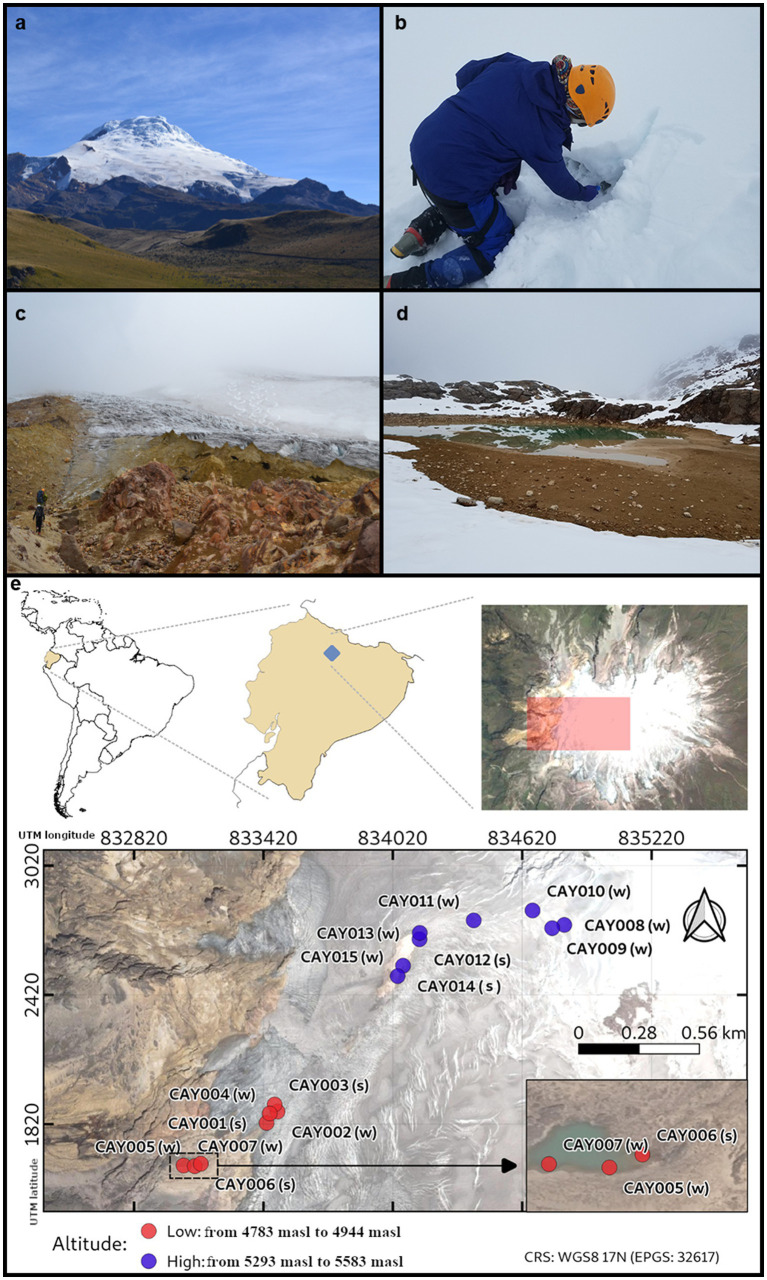
The Cayambe Volcanic Complex (CVC). A view of the western face of the CVC, including its glacier **(A)**. The first author sampling ice **(B)**. Researchers on their way to the CVC glacier **(C)**. A view of the lake called “Laguna Verde” where some samples were obtained **(D)**. Location of the CVC and map of the collected samples along the glacier ascension route (red and purple dots) **(E)**. Samples were categorized into high-altitude (purple, from 5,293 masl to 5,583 masl) and low-altitude (red, from 4,783 masl to 4,944 masl) and into glacier soil (s) and glacier ice (w). Samples were labeled in correspondence to Supplementary Data 1.

The Cayambe Volcanic Complex (CVC) is a massive explosive volcanic center with a base extension of 24 × 18 km. It rises to an altitude of 5,790 M above sea level (masl), and it is covered by a vast ice cap of nearly 22 km^2^, with a thickness that reaches up to 100 m in specific areas and an approximate volume of 0.7 km^3^ (Monzier et al., 1996; Guillier and Chatelain, 2006; Figure 1). The CVC ice cap is present above 4,800 masl and descends to ~4,600 masl on its western flank and ~ 4,200 masl on its eastern flank (Samaniego et al., 1998; Detienne et al., 2017; Bax and Francesconi, 2019). The glacier retreat of the CVC has been estimated at 25.58% from 1979 to 2009 (Gallegos Castro et al., 2018). The CVC is unique in its geographical location, which is essentially at zero latitude (0.03° N; 77.988° W). During the last 4,000 years, the CVC has experienced 21 volcanic eruptions, the most recent occurring in 1785–1786 (Samaniego et al., 1998). The glacier of the CVC serves as a source of water for surrounding communities, including large cities such as Quito.

Microbial communities should be perceived not only as the presence and interactions of microscopic living organisms but also as the biological matrix which plays a vital role in shaping ecosystems and communities of multicellular organisms (Stolz, 2017). Microbial communities at mountain glaciers are often first colonizers and key players in soil formation, which enable subsequent processes of plant colonization and growth, transformation of compounds, rock weathering and nutrient enrichment of downstream ecosystems (Ragot et al., 2013); yet, it is unknown, particularly for the Andes, which are the consequences of rapid glacier melting, due to climate change, on the microbial communities and their ecological function (Ciccazzo et al., 2016).

Substantial amounts of biodiversity for multicellular organisms are well known for the tropical Andes (Bax and Francesconi, 2019); however, there are still few studies on microbial diversity for the region, particularly at glaciers and high-altitude mountain environments (Ciccazzo et al., 2016; Hotaling et al., 2017; Nayfach et al., 2020). Most of the studies of microbial communities at mountain glaciers come from the European Alps or the United States; thus, information from the Neotropical Andes is needed for a broader vision of climate change effects and ecological processes on a global scale (Ciccazzo et al., 2016). These studies have shown that: (1) microorganisms play a crucial role in soil formation from glacier rock and biogeochemical cycles, enabling the arrival of first multicellular colonizers; (2) their physiology is largely influenced by physicochemical and environmental factors such as pH, moisture, and temperature; (3) their communities can be structured as a function to distance from the glacier terminus and soil chronosequence; and (4) glaciers are capable of maintaining specialized communities of psychrophilic microorganisms that often show upregulation of genes for cold-shock proteins and exopolymers (EPS; Ciccazzo et al., 2016). However, all these aspects have been found and described in glaciers located at other latitudes than the tropics and it remains to be seen if such general principles apply to these other environments (Ciccazzo et al., 2016; Hoham and Remias, 2020).

A thorough assessment of microbial diversity in the Andes is crucial to establish the potential for further prospection into the use of psychrophilic microorganisms and derived bioproducts of microbial metabolism (Borda-Molina et al., 2017). Environmental services, as the result of bacterial metabolism, are also an important reason why we need to understand bacterial communities in these fragile and rapidly changing environments (Margesin et al., 2009). Bacterial communities from extreme glacier environments have been evaluated by applying next-generation sequencing of the 16S RNA region in substrates such as glacier soil and glacier ice (Schloss, 2020), without the requirement for cultivation (Tan et al., 2015; Chan et al., 2019).

Our objective was to investigate the structure and distribution of bacterial communities in the CVC, which is a poorly understood ecosystem at risk of significant alterations due to climate change. Additionally, we aimed to explore the relationship of physicochemical environmental variables with these bacterial communities. Although the manuscript primarily focuses on the structure and distribution of bacterial communities, we have also analyzed the potential influence of environmental factors on these communities. Accessing Andean glacier ecosystems such as the CVC is a challenging endeavor. Along the ascension route to the summit of the CVC, we found that the environment is a patchy combination of two main types of substrates, glacier soil and glacier ice; thus, our assessment includes substrate as a major component on the analysis. Glaciers run the risk of disappearing and with them their evolved microbiomes (Staley, 1997). Recording the most remarkable aspects of these endangered psychrophilic microbial communities is essential to understand the potential losses for biodiversity and how this may further impact the environment (Peter and Sommaruga, 2016).

Based on the arguments exposed by Ciccazzo et al. (2016), we hypothesized that elevation would be a significantly correlated component to differences in the composition of the observed communities. We also hypothesized that these differences will be linked to significant correlations in the concentration of nutrients and other physicochemical properties (as described in the methods section) that are relevant for bacterial life.

## 2. Materials and methods

### 2.1. Sample collection and environmental analysis

Samples were collected on November 28th, 2015 (Figure 1). The chosen route provided an opportunity to gather samples from both glacier soil and glacier ice, which allowed for an additional level of contrast in the context of elevation effects and substrate physicochemical properties on bacterial diversity. A shovel or ice axe was used to dig into the sampling point at an approximate depth of 10–25 cm below the surface, removing rocks. Samples were taken in duplicate with a shovel previously washed and disinfected with 70% alcohol and immediately stored in hermetically-sealed sterile plastic bags. To avoid sample contamination during sample collection, we followed the recommendations provided by EPA (EPA, 2020a,b). Each time a different sample was collected, a pair of new, non-powdered disposable gloves were worn. The gloves were not in contact with the sampled substrates and were changed each time a new sample was obtained. Plastic bags and sample containers were new, disposable, and sterilized by UV irradiating prior to sampling. Glacier soil samples consisted of 1 kg of material. Glacier ice samples consisted of 1 l of ice. Samples arrived in a cooler box to the laboratory after 8 h of being collected and stored in a 0°C freezer. Each sample was used for the extraction of total genomic target DNA and the determination of physicochemical properties.

Starting at 4,800 masl, soil becomes increasingly less visible as it is covered by glacial ice along our ascension near the summit at 5,600 masl. As a visual aide to the nature of samples, we have labeled glacier soil (s) and glacier ice (w) in the representation provided by Figure 1. Samples were labeled by the letters “CAY” and followed by serial numbering. Along the ascension route to the summit of the volcano, soil became increasingly less accessible, as it was covered by glacial ice. An interval of difficult access for sampling created two groups of samples that were separated by elevation: these were low-altitude samples (from 4,783 masl to 4,944 masl) and high-altitude samples (from 5,293 masl to 5,583 masl; Figure 1). The elevation gap between these two groups of samples corresponded to 349 masl and was the result of difficult terrain that precluded establishing a regular path of collection points. Given this gap in elevation between the two groups of samples, we expected to find differences in the estimated community composition among them.

Informed by previous studies on bacterial communities (Singh et al., 2014; Looby et al., 2016; Peay et al., 2017; Nottingham et al., 2018), we chose a set of physicochemical properties to measure and describe the obtained samples. These were analyzed at the Center for Integral Analytical Solutions (CENTROCESAL Cía.Ltda., Ecuador. Accreditation No SAE LEN 12–001) and consisted of the following 18 parameters: electrical conductivity (EC) (μsiemens/cm), organic matter content (Org) (%p/p), total hardness (TH) (mg/L), humidity (%p/p), cation exchange capacity (CEC) (meq/100 g), phosphate (PO_4_^3−^) (ppm), nitrogen (N) (ppm), calcium (Ca^2+^) (ppm), magnesium (Mg^2+^) (ppm), manganese (Mn^2+^) (ppm), sulfate (SO_4_^2−^) (ppm), potassium (K^+^) (ppm), sulfur (S) (ppm), iron (Fe^3+^) (ppm), sodium (Na^+^) (ppm), chloride (Cl^−^) (ppm), calcium carbonate (CaCO_3_) (ppm), and total dissolved solids (TDS) (ppm). These parameters were obtained according to the procedures described in (Baird et al., 2017). pH was evaluated *in situ* with a portable pH meter (Mettler-Toledo SevenGO, Millipore, Columbus, OH, United States). Data from the physicochemical analyses are included in Supplementary Data 2, 3. In conformance to the ISO/IEC 17025:2017 competence of testing and calibration laboratories standard, a minimum of two samples was always employed for each soil chemical measurement.

### 2.2. DNA extraction, 16S rRNA gene library preparation, and sequencing

Total glacier soil genomic DNA was isolated with the PowerSoil DNA Isolation kit (Cat. No. 12888-50, MoBio Laboratories, Inc., Carlsbad, CA, United States). Total glacier ice (glacier) genomic DNA was isolated with the PowerWater DNA Isolation kit (Cat. No. 14900-50 NF MoBio Laboratories, Inc.). The total extracted genomic DNA is currently stored at −80°C in the collection of the Ecuadorian Microbiome Project (EcMP) at the Institute of Research on Zoonoses (CIZ) of Central University of Ecuador. A partial region of 500 bp including the hypervariable regions V3 and V4 of the 16S rRNA genes was amplified with custom primers based on previous work (Klindworth et al., 2013). The primer pair was: forward = 5’-TCGTCGGCAGCGTCAGATGTGTATAAGAGACAGCCTACGGGNGGCWGCAG-3′ and reverse = 5’-GTCTCGTGGGCTCGGAGATGTGTATAAGAGACAGGACTACHVGGGTATCTAATCC-3′. 16S rRNA libraries of 300 bp paired-end fragments of the bacterial metagenome were obtained by synthesis sequencing technology on an Illumina MiSeq platform (San Diego, CA, United States). The studied sequences are available at NCBI with the BioProject accession number PRJNA681925. We included two types of negative controls (Kim et al., 2017; Eisenhofer et al., 2019). First, a blank extraction control was included during DNA extraction and all subsequent protocol steps. This blank control had no input material. Second, we included a blank library control, in which the extraction protocol was not applied and DNA-free water was used as input to library generation and further sequencing.

### 2.3. Sequence processing and analysis

Prior to performing taxonomic annotation, all sequence files were checked for quality with FastQC (Andrews, 2010). The identification of bacterial groups was assisted by Mothur v.1.43.0 (Schloss et al., 2009) and according to the MiSeq Standard Operational Procedure (Kozich et al., 2013). Forward and reverse reads were assembled into contigs and the resulting sequences were filtered and processed. We retained sequences with a minimum overlapping of 20 bp, a maximum length of 580 bp, and a minimum of 348 bp. Sequences with homopolymers longer than 14 bp or containing ambiguities were also removed from the analysis. The filtered sequences were deduplicated and aligned against the V3-V4 region of the SILVA v132 reference small subunit rRNA gene alignment database. Those sequences that did not span the full alignment were filtered by optimizing the start and end positions using a 95% criterion. The alignments were processed by eliminating columns that exclusively contained gaps or dot characters, and the sequences were deduplicated for a second time. Denoising was performed by preclustering sequences with less than one difference per 100 bp, and chimeras were removed using Mothur’s implementation of the VSEARCH algorithm (Rognes et al., 2016). Sequences were classified with a naive Bayesian classifier against the SILVA v132 reference taxonomy database, by the Wang method (Wang et al., 2007) and with a 70% bootstrap threshold. Sequences belonging to chloroplasts, mitochondria, and Eukaryota were removed. The final resulting sequences were clustered into OTUs at 99% identity with the opticlust algorithm (Westcott and Schloss, 2017). The most abundant sequence within each sequence cluster served for consensus classifications and the determination of representative sequences for each OTU. All the commands used in the Mothur pipeline for sequence processing are available in the file “Mothur_v1.43_V3V4_DEF.batch” at.^1^ Processed Mothur data were imported into R (R Core Team, 2020) with the phyloseq package (McMurdie and Holmes, 2013). OTUs were grouped at the genus and family levels, and taxonomic levels kingdom and phylum were inspected to filter Archaea/unknown taxa and unclassified bacteria, respectively. Genera with zero counts in all samples were also removed. Bacterial composition was explored at various taxonomic levels with plots generated in Krona (Ondov et al., 2011). Afterward, samples were separated by substrate (soil and water-ice), removal of singletons was performed, and the subsequent analyses were carried out.

### 2.4. Diversity analysis

Diversity indices were estimated for each sample site, including Chao, Shannon, and Simpson. α-diversity was compared across sample sites and the two categories of altitude (high vs. low) with a one-sided Wilcoxon signed-rank test. To test the relationship of α-diversity and altitude, a robust linear regression by an iterated re-weighted least squares model was applied with the Chao index as the dependent variable and altitude as the regressor. This was applied through the “rlm()” function in the MASS package in R (Venables and Ripley, 2002). Following the estimation of the slope in the regression model, we tested its significance through a Wald test (or robust F-test) through the “f.robtest()” in the sfsmisc package in R (Maechler, 2022).

Rarefaction curves with steps of 600 samples for soil and glacier ice were estimated with the back-end functions of the ranacapa package (Kandlikar et al., 2018). Heatmaps of the log-transformed counts were used to visually compare the overall absolute abundance between samples at the family level, the community structure in individual samples, and the metacommunities in soil and glacier ice. To avoid overplotting, only the most abundant families were selected for each sample and based on the log count transformation; for glacier ice sequences, the cutoff was log(x + 1) > 25.3 and for soil sequences, the cutoff was log(x + 1) > 15.6. With the selected families, a hierarchical clustering, with the unweighted pair group method (UPGMA on Euclidean distances), was performed to evaluate if this analysis could capture the change in community composition across the altitudinal gradient (Gu et al., 2016).

The patterns provided by the abundance heatmaps could be summarized in the concept of γ-diversity, with the added benefit of robust estimation of entropy to a meaningful measure of biological diversity (Jost, 2006; Marcon and Hérault, 2015). To test for differences in the structure of the meta-community in glacier soil versus glacier ice, we obtained a corrected estimate distribution of the γ-Shannon diversity; package entropart (Marcon and Hérault, 2015) was used for this purpose.

### 2.5. Ordination and differential abundance analyses

Underrepresented genera were removed based on the arbitrary threshold criteria that genera had to be detected at least five times in more than half of the samples; additionally, only the five most abundant phyla were kept since they represented over 90% of the relative abundance. Genera count data were transformed to even sample depth by multiplying a constant by the relative abundance. The constant value was the average sample depth for glacier ice (i.e., 48,741) and glacier soil samples (i.e., 50,410) respectively. The filtered phyloseq object (previously explained in the sequence processing and analysis section) was exported to a DESeq2 object for further preprocessing (Love et al., 2014). Based on the transformed DESeq2 object, the size factors of the abundances were estimated through the median rate method (Anders and Huber, 2010). The abundances in the DESeq2 object were subjected to variance stabilizing transformation by using the estimated size factors.

β-diversity was assessed through a non-metric multidimensional scaling analysis (NMDS) with fitted environmental (physicochemical) variables. The algorithm for fitting environmental variables to the NMDS space found the direction in which the correlation of the environmental vectors was the strongest; the associated statistical significance in this context was for a null hypothesis in which the correlation was indistinguishable from zero (Oksanen et al., 2018). The NMDS was based on Bray-Curtis distances, which were obtained from the original matrix of abundances for families across samples. We used a radar plot to show the distribution of scaled physicochemical variables for glacier ice and soil samples and grouped them by two categories of altitude (low vs. high). The intersection of bacterial families in the two categories for altitude (high vs. low) and substrate (soil vs. glacier ice) were depicted in a Venn diagram. Families used in the Venn diagram were those present at least five times in more than half of the samples. NMDS analyses were made with the vegan package (Oksanen et al., 2018).

To discover significant differences in the presence of genera between low- and high-altitude communities, a differential abundance detection analysis, based on a negative binomial distribution, was performed with the DESeq2 package (Love et al., 2014). This analysis returned the computed log2 fold change and corresponding *p*-values. The latter was corrected by the Benjamini-Hochberg method (Benjamini and Hochberg, 1995), as a threshold to minimize the false discovery ratio. Genera were projected into a volcano plot, with –log_e_(*p*) against the log2 fold change. Since the fold change was obtained by low-altitude/high-altitude abundance ratios, those genera with a positive fold change will express larger abundance at low altitudes, and those with a negative fold change will express larger abundance at high altitudes. The abundance distribution of all families that were common to all samples, irrespective of the type of substrate, provided a perspective on the meta-community. This pattern was represented by a heatmap of the log-transformed counts and an accompanying cluster analysis with the unweighted pair-group method and based on Euclidean distances. The statistical procedures are available at (see footnote 1).

## 3. Results

A total of 15 samples were obtained from the CVC, which included a range from 4,783 to 5,583 masl (Figure 1). Coordinates and altitude for each sample are included in the Supplementary Data 1. A total of 252,053 16S amplicon high-quality reads were obtained for glacier soil samples, with an average of 50,410 ± 15,468 reads per sample. A total of 487,414 16S amplicon high-quality reads were obtained for glacier ice samples, with an average of reads per sample of 48,741 ± 12,976. The available sequence samples were classified into 1,037 genera.

We recorded a total of 41 phyla, with *Proteobacteria*, *Actinobacteria*, *Bacteroidetes*, *Acidobacteria*, and *Firmicutes* common to all samples. The three most abundant phyla in glacier soil samples were *Actinobacteria*, *Proteobacteria*, and *Acidobacteria*. In glacier ice samples, the three most abundant phyla were *Actinobacteria*, *Proteobacteria*, and *Bacteroidetes*. For either glacier soil or glacier ice, these four phyla constituted up to 75% of the relative abundance. On average, the predominant phylum in glacier soil was *Actinobacteria*, with 25 and 34% of total sequences at high and low altitudes, respectively, (interactive Krona plot of all taxonomical categories found in glacier soil available at).^2^ In contrast, for glacier ice samples, *Proteobacteria* was the predominant phylum at high altitudes (47%) and was replaced by *Actinobacteria* as the most abundant at low-altitude samples (43%) (interactive Krona plot of all taxonomical categories found in glacier ice samples available at).^3^ Individually, *Proteobacteria* was the richest phylum in the CVC with 11 families, followed by 8 families in *Actinobacteria*, 5 in *Firmicutes*, 4 in *Bacteroidetes*, and 1 in *Acidobacteria*.

**Figure 2:**
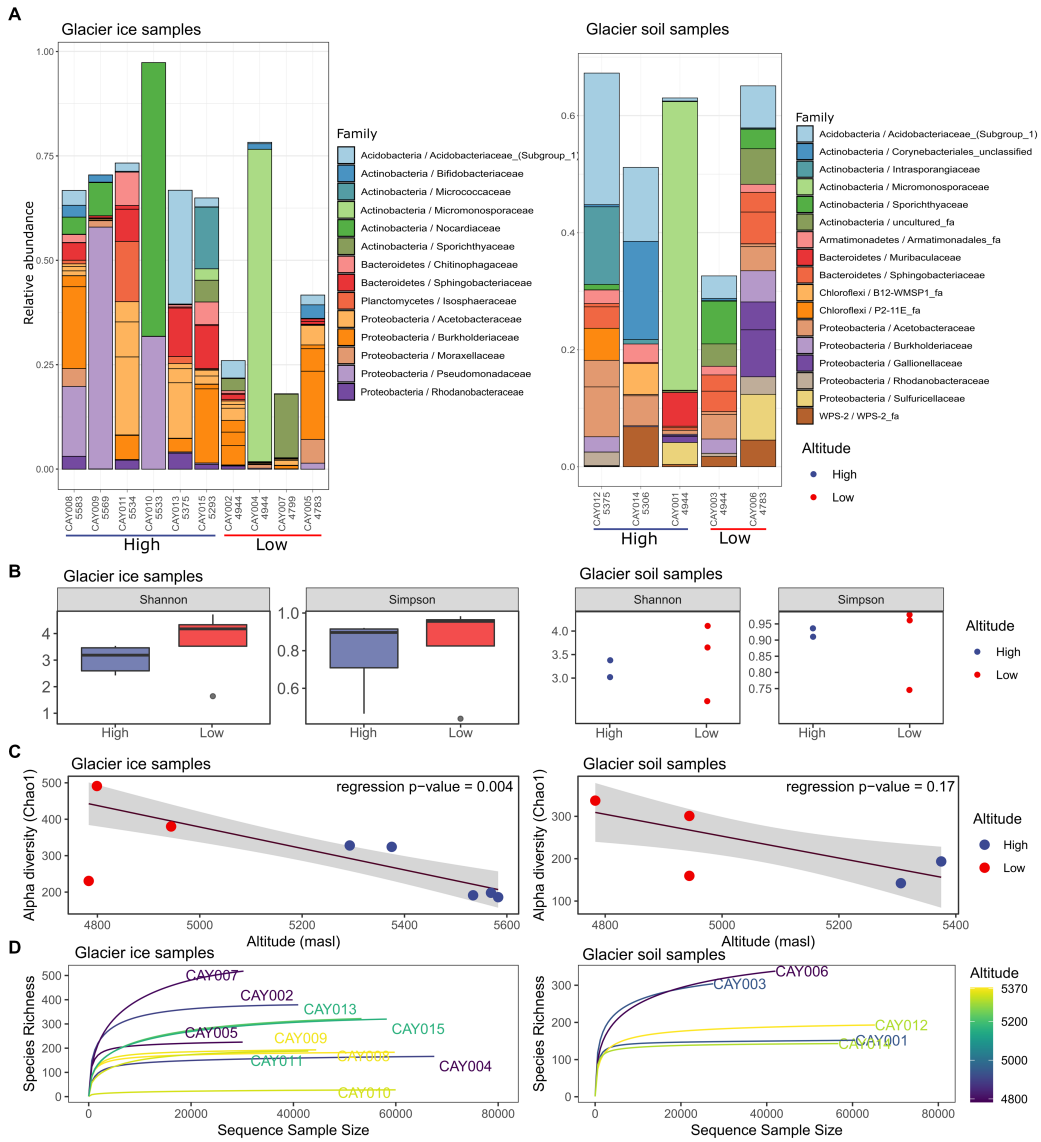
Community α-diversity analysis of the glacier ice (left column) and glacier soil (right column) microbiomes. The stacked bar plot depicts the relative abundance for the 20 most abundant families in all samples and was generated on all recorded families **(A)**. Shannon and Simpson diversity measurements for glacier ice and soil samples and a comparison between the two categories of altitude; boxplots were not possible for soil samples due to small sample size **(B)**. Robust linear regression with the Chao1 diversity index as the response variable and altitude as the regressor, it includes a 0.95 confidence interval as a shaded area **(C)**. Rarefaction curves for glacier ice and soil samples **(D)**.

Glacier soil and glacier ice samples shared half of the 10 most abundant families. Some samples showed the presence of a single superabundant family (>50% relative abundance), such as CAY004 (4,948 masl, Micromonosporaceae 79%), CAY009 (5,569 masl, Pseudomonadaceae 58%) and CAY010 (5,533 masl, Nocardiaceae 66%) for glacier ice samples, and CAY001 (4,945 masl, Micromonosporaceae 52%) for glacier soil samples (Figure 2A and interactive Krona plots for soil and glacier ice samples). There was no discernable pattern or relationship between samples and their geographical location to explain the dominant presence of these families (Figures 1, 2).

There was a trend toward higher diversity at lower altitudes for both glacier ice and glacier soil (Figures 2B,C). A robust linear regression on the Chao1 diversity index, with altitude as the regressor, showed a markedly inverse relationship for glacier ice samples (*F* = 20.27, *p* = 0.004), but not for glacier soil samples (*F* = 3.23, *p* = 0.17; Figure 2C); the latter showed no statistical significance. A one-sided Wilcoxon signed-rank test, comparing the diversity of glacier ice samples from high altitudes vs. those from low altitudes, showed significance (*W* = 0, *p* = 0.018 for the contrast on the Shannon index and *W* = 0, *p* = 0.036 for the contrast on the Simpson index). However, the same test performed in glacier soil samples provided no significance (*W* = 0, *p* = 0.17 for the contrast on the Shannon index and *W* = 0, *p* = 0.17 for the contrast on the Simpson index; Figure 2A). All rarefaction curves for richness approached an asymptote within at least 60% of reads, which indicated a sufficient sequencing depth (Figure 2D).

**Figure 3:**
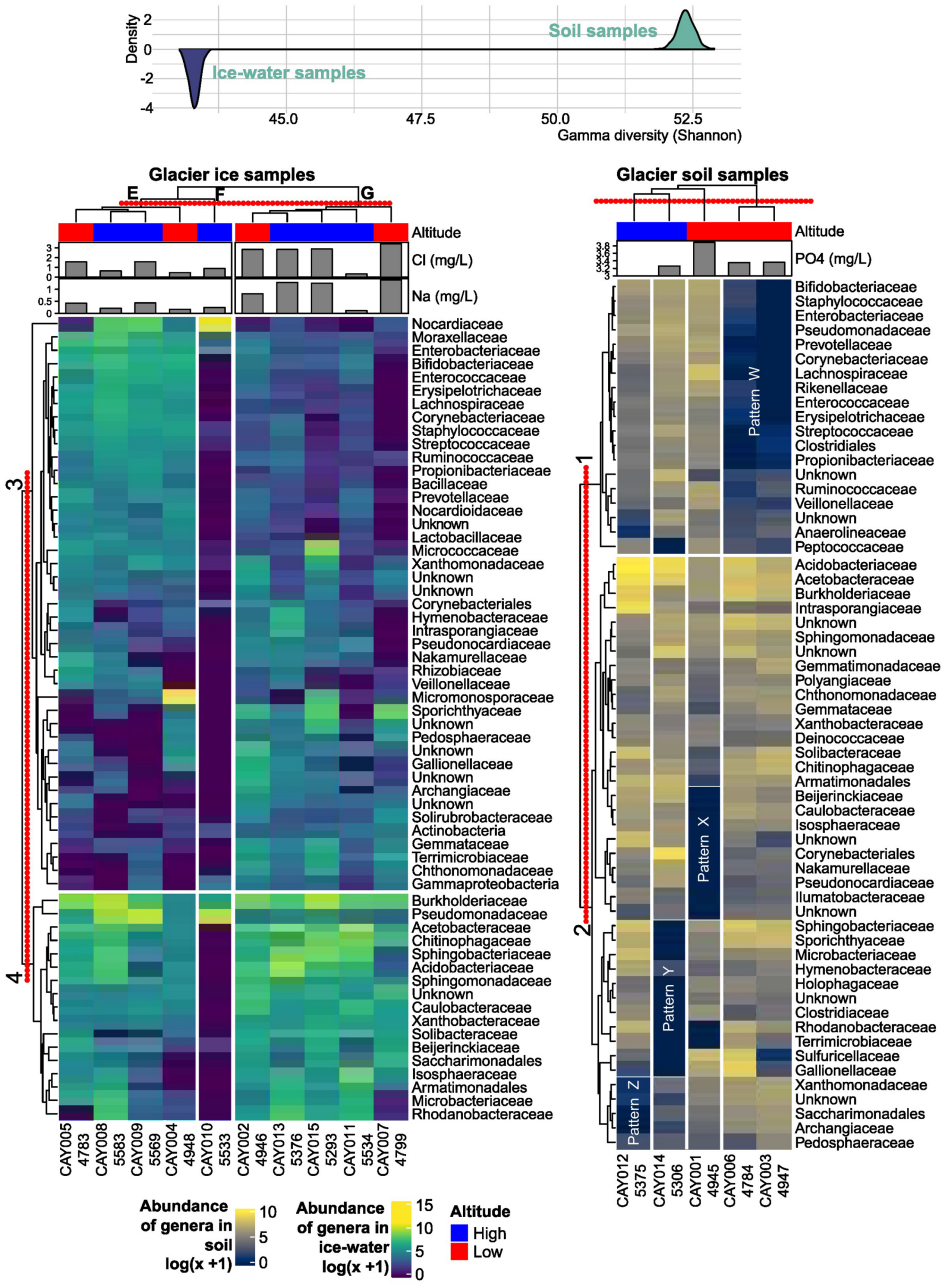
Abundance heatmaps of the most abundant families and hierarchical clustering. The concentration of phosphate (PO_4_^3−^), sodium (Na^+^), and chloride (Cl^−^) are included above each heatmap, as these physicochemical variables showed a significant correlation with the distribution of samples in an NMDS analysis. The red-dotted line above each cluster represents the distance at which groups are defined. Altitude is included for each sample below its name. Above the heatmaps is the estimated Shannon γ-diversity distribution for either soil or glacier ice metacommunities. The latter distribution has been inverted to accentuate its contrast to the former.

A complex pattern of abundance in the samples can be summarized by the heatmap on the most abundant families and its interpretation was assisted by the accompanying clustering (Shannon γ-diversity distribution). For the interpretation of the observed patterns, clusters for families (along the rows or horizontal direction) were numbered from 1 to 4, and clusters for samples (along the columns or vertical direction) were labeled from A to G. For glacier soil, two clusters of families were established. Within Cluster 1 there was a sharp difference between the sample cluster formed by CAY006 (4,784 masl) and CAY003 (4,947 masl) (cluster D) and the rest of the samples in clusters A, B, and C. This difference highlighted a remarkable correspondence between the clustering results of samples and the clustering results of bacterial families, which pointed toward strongly structured communities in glacier soil. Although highly similar in the abundance of families in Cluster 1 (pattern W in Figure 3), CAY003 and CAY006 were separated by approximately 500 m, and each one was closer to other, less similar, sampling sites (Figures 1–3). Both CAY003 and CAY006 belonged to the low-altitude glacier soil sample category. Sample CAY001 (4,945 masl), which formed cluster C, was characterized by the marked low abundance of the families in Pattern X (Figure 3). Similarly, samples CAY0012 (5,375 masl) and CAY0014 (5,306 masl) were characterized by Patterns Z and Y respectively, which showed conspicuously low abundance for different groups of families (Figure 3). Sample groups A, B, C, and D in glacier soil had all conspicuous patterns of abundance for different groups of families (i.e., patterns W, X, Y, and Z in Figure 3). The clustering results for soil samples in the heatmap suggested an effect of altitude on the structure of communities.

In comparison to the glacier soil samples, glacier ice samples showed less structure or recognizable patterns in terms of the observed abundance in families. In other words, there was more homogeneity among the communities in ice than in soil. Sample CAY010 (5,533 masl), which formed cluster F, can be easily differentiated by the presence of low abundance in most families when compared to the rest of glacier ice samples (Figure 3). Notably, Nocardiaceae, which is an actinomycetes family found also in Antarctica (Roslee et al., 2020), was uniquely abundant in CAY010.

Sharp differences in abundance for different groups of families within glacier soil samples, in comparison to the more homogeneous distribution of abundance in glacier ice samples, was a pattern that was summarized in terms of γ-diversity. The latter contrast showed sharp differences between the two types of substrates, with the simulated distributions having no overlap and separated by at least 8 units of γ-diversity (Figure 3).

**Figure 4:**
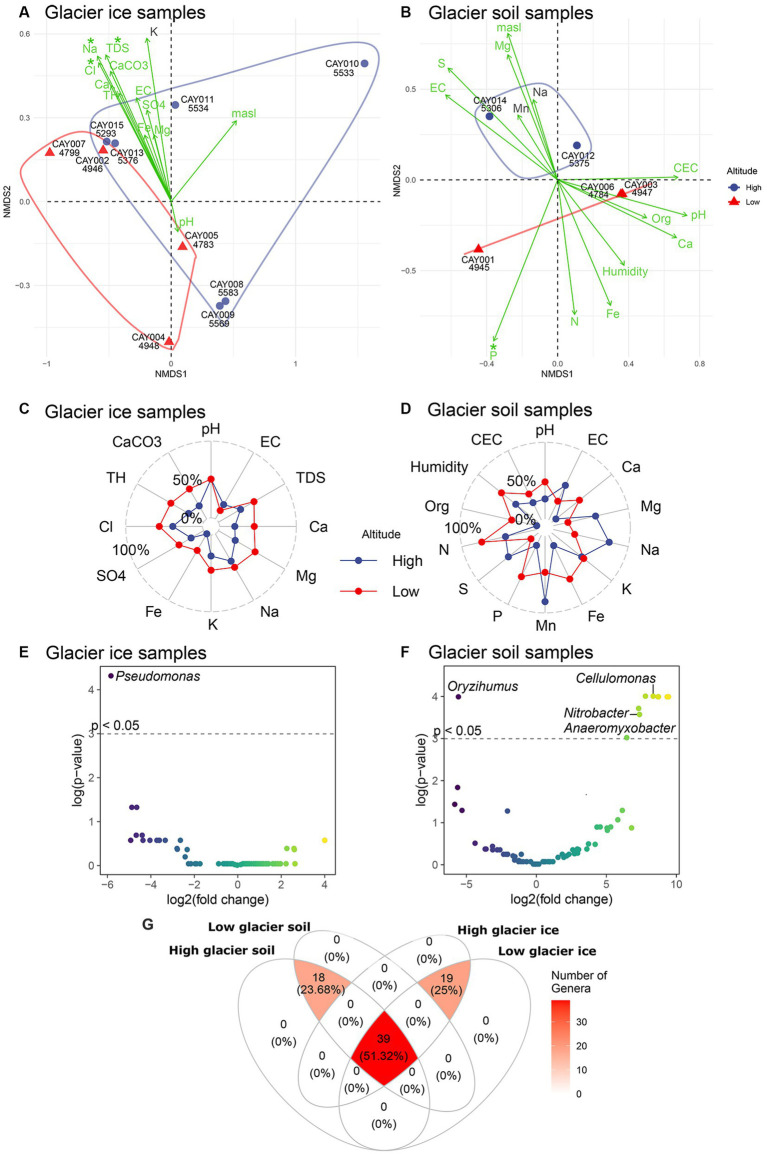
Ordination and differential abundance analysis. An NMDS for soil **(A)** and glacier ice **(B)** samples, with physicochemical variables as fitted vectors. Asterisks over the name of each environmental vector show significance (*p* < 0.05) for the correlation with the scores of samples. Samples are represented by color according to high-altitude (blue) or low-altitude (red). A convex hull around samples has been included to facilitate the contrast between the two categories of altitude. The radar plots for soil **(C)** and glacier ice **(D)** samples show average differences (as percentages) in the concentration or magnitude of physicochemical variables between high- and low-altitude samples. The volcano plots show the results of the differential abundance analysis at the genus level for glacier ice **(E)** and soil **(F)** samples. The cutoff to minimize the false discovery rate was set to *p* < 0.05 and is represented by the dashed horizontal line. The color of the data points varies accordingly to the intensity of the log2 fold change. A Venn diagram of the shared families between substrates and categories of altitude, only families that were detected more than five times in at least half of the samples were included **(G)**.

Low-altitude communities were different in composition from high-altitude communities in glacier soil samples, but not in glacier ice samples (Figures 4A,B). The differences in soil communities were evident along the second axis of the non-metric multidimensional scaling analysis (NMDS; Figure 4B), but glacier ice samples showed considerable overlap on either the first or second axis of the NMDS (Figure 4A). In glacier ice samples, the largest fitted environmental vectors (i.e., highly correlated environmental variables to sample scores) were chloride, sodium, and total dissolved solids, which were also the only significant ones (*p* ≤ 0.05). These three environmental vectors were strongly and significantly correlated to the distances among samples in the NMDS space, and therefore to community structure, but did not contribute to differences between the two categories of altitude (Figure 4A). In glacier soil, one of the largest fitted environmental vectors was phosphate and the only one with significance (*p* < 0.05). The separation of high-altitude vs. low-altitude glacier soil samples was therefore correlated with a gradient of concentration in which phosphate was higher at lower altitudes (Figure 4B). Circumstantial evidence was present for differences in the concentration or magnitude of several physicochemical parameters between high- and low-altitude samples; however, due to the small sample size available, no contrast showed statistical significance (Supplementary Figure S1). When compared to high-altitude glacier ice samples, low-altitude glacier ice communities had a higher concentration or larger values for all physicochemical variables, except for electrical conductivity (EC; Figure 4C). A more complex pattern was present for glacier soil, in which magnesium, sodium, manganese, and sulfate had larger concentrations at higher altitudes, and pH, organic matter, nitrogen, iron, calcium, and phosphate had larger concentrations at lower altitudes (Figure 4D).

There was a remarkable and significant change in abundance (*p* < 0.05) for *Pseudomonas* between low- and high-altitude glacier ice communities as this genus was strongly (i.e., effect size) and significantly more abundant at higher elevations (Figure 4E). For glacier soil, the genus *Oryzihumus* was strongly and significantly more abundant at higher elevations (Figure 4F); on the other hand, significantly and strongly less abundant at higher elevations were *Nitrobacter, Cellulomonas,* and *Anaeromyxobacter,* plus five additional unidentified genera (Figure 4F). About half (51.32%) of the 76 families found in this study were present in all the combinations of altitude and substrate; 18 families were common to all soil samples, irrespective of the altitude category, and 19 families were common to all glacier ice samples irrespective of the altitude category (Figure 4G). Remarkably, neither the substrate categories (i.e., glacier soil or glacier ice) nor the altitude categories (high or low) presented exclusive families, as all the 76 families were shared between categories (Figure 4G).

## 4. Discussion

Our study encompassed a gradient of elevation and two substrate groups (i.e., glacier soil and glacier ice). We found a difference in α-diversity along the elevation gradient for glacier ice, where low-altitude communities (<5,200 masl) presented higher α-diversity than high-altitude communities (>5,200 masl). However, glacier soil showed no effect of altitude on α-diversity. Correlations between elevation and diversity in microbial ecology can mask several underlying ecological and physicochemical parameters (Lanzén et al., 2016). Previous studies have found environmental parameters that were significantly independent of elevation, and where the latter factor was secondary to other parameters in explaining the structure of bacterial communities (Fierer et al., 2011; Díaz et al., 2022). Although we acknowledge the possibility of confounding or unaccounted factors that could be underlying elevation as a significant component for bacterial diversity, such as soil moisture, soil nutrient status, substrate availability, and substrate quality, (Meier et al., 2010; Nottingham et al., 2015), we have also included as part of our assessment a set of 18 physicochemical parameters, whose correlations with the observed community diversity and structure are discussed in the next paragraphs.

Our results are consistent with earlier studies on microbial diversity along a mountain elevational gradient (Lanzén et al., 2016; Shen et al., 2020). Decreasing α-diversity with higher altitude was also reported for bacterial communities in mountain glaciers from the Austrian Alps (Wilhelm et al., 2013), the Tianshan Mountains in Central Asia (Ren et al., 2017), and the Himalayas (Liu et al., 2011). Schütte et al. (2010), in a glacier foreland of the High Arctic, reported constant levels of diversity for different samples, irrespective of the chronosequence (i.e., glacier retreat). On the other hand, Schmidt et al. (2009) found that diversity increased along lower elevations from a receding glacier in southeastern Peru. Increments in biodiversity at lower glacial altitudes have been reported not only at the prokaryotic scale but also for macroinvertebrates and other groups of multicellular organisms (Milner et al., 2001; Jacobsen and Dangles, 2012; Cauvy-Fraunié and Dangles, 2019). A recent synthesis on the effect of altitude on soil bacteria diversity can be found in Díaz et al. (2022), which shows that the issue is currently not fully understood and lacks universal consensus.

Our results conform to the possible effect of soil as a promoter of diversity and specialization in bacterial communities and its contrast to glacier ice environments. The alternating pattern in the radar plot for soil, where the means across samples of physicochemical parameters are not homogeneously distributed between altitudinal categories as they were in glacier ice (Figures 4C,D; Supplementary Figure S1), points to a more complex ecosystem in soil.

Five phyla were found to be common to all samples: *Proteobacteria*, *Actinobacteria*, *Bacteroidetes*, *Acidobacteria*, and *Firmicutes*. This finding was consistent with the most abundant phyla previously reported in glacier environments (Simon et al., 2009; Xiang et al., 2009; Schütte et al., 2010; Jacobsen and Dangles, 2012; Seok et al., 2016). The occurrence of these psychrophilic phyla in other glacier ecosystems was also validated by culture-dependent methods (Cheng and Foght, 2007; Loveland-Curtze et al., 2009). *Acidobacteria* has been found as one of the most abundant phyla in glacier soils, but not in water (Lee et al., 2013; Park et al., 2015). The same trend was determined in our study, where *Acidobacteria* was the third most abundant phylum in glacier soil communities. The occurrence of superabundant families, such as Micromonosporaceae in sample CAY004 (4,948 masl), may be related to competitive exclusion, as antibiotic-producing bacteria may dominate over the rest of the species in the community. Members of Micromonosporaceae are a well-known source of antibiotics (Talukdar et al., 2016).

EC and pH have been reported as important environmental variables that may affect the microbiome in glacier water since these factors have a notable physiological effect on single-celled organisms (Brown et al., 2007; Wilhelm et al., 2014). However, pH and EC were not significant variables to explain the community structure in our survey of the CVC. We propose that significance was absorbed by other factors involved in EC such as the higher presence of salt ions in low-altitude glacier ice samples (Na^+^ and Cl^−^) and which coincide with the general direction of the EC vector in the ordination analysis (Figure 4A).

Other studies have shown that EC is greater at lower altitudes from the glacier summit (Milner et al., 2001, 2009). In the case of the CVC, we did not find evidence for a relation between EC and altitude or the composition of communities; however, Cl^−^ and Na^+^, considered here as a proxy for EC, were strongly correlated to an observed pattern of community composition in glacier ice samples, in which a mixture of low- and high-altitude communities were clustered together (Figures 3, 4A). EC has been proposed as a driver for diversity in glacier ecosystems, as liquid water at lower altitudes may be linked with higher magnitudes of this parameter (Wilhelm et al., 2013). A negative correlation between altitude and EC has been reported for soil matrices at other study sites (Calvo et al., 2009; Wu et al., 2015). This may be related to higher concentrations of nutrients downstream, as rain and meltwaters flow down the glacier toward lower elevations, water may carry minerals and mobilized ions, which will enrich lower-elevation substrates and environments (Ciccazzo et al., 2016). Phosphorus has been considered as a limiting elemental resource for soil bacterial communities (Ragot et al., 2013); thus, this was the only variable (measured as PO_4_^3−^) with a significant correlation to the observed bacterial community composition in glacier soil, and with higher concentrations at lower elevations (Figures 3, 4). The bioavailability of phosphate may play an important role in shaping bacterial communities at Andean glacier environments.

### 4.1. Differential abundance analysis

Selection pressures and living conditions in glacier ice are more demanding for unicellular organisms than other kinds of substrates such as soil (Ciccazzo et al., 2016; Cazzolla Gatti et al., 2018); thus, when compared to glacier ice, the glacier soil had more structured, diverse, and specialized communities, as measured by γ-diversity (Figure 3). The differential abundance analysis, between low- and high-altitude samples, found at least four significant genera in glacier soil (*Nitrobacter*, *Cellulomonas*, *Oryzihumus*, and *Anaeromyxobacter*), but only one for glacier ice (*Pseudomonas*). The latter pattern may be related to markedly structured communities in glacier soil when compared to glacier ice. These salient genera detected by the differential abundance analysis could be proposed as biomarkers for the detection of either low- or high-altitude substrate samples and the effects of the receding glacier on the composition of bacterial communities.

*Pseudomonas* is a genus with psychrophilic species (Margesin et al., 2009), such as those in the *Pseudomonas fluorescens* complex (Mukhia et al., 2022). Species in this complex are capable of ice-nucleating activities (Obata et al., 1998). Within the *P. fluorescens* complex, there is a group named *P. antarctica* (Vásquez-Ponce et al., 2018), which consists of Antarctic species, but has also been reported from the East Rathong supraglacial site in Sikkim Himalaya (Mukhia et al., 2022). We found that this genus was significantly and strongly more abundant at higher altitudes (>5,200 masl) in glacier ice communities. Metabolic results of isolated bacteria from CVC using dedicated culture-dependent methods (E. Rivadeneira, unpublished) and whole metagenome analysis are expected to elucidate the relevance of this group of microorganisms for glacial ecosystems. It is noteworthy that although the differential abundance analysis with glacier soil samples found 10 significant genera (two points are overlapping in Figure 4F), only four of them were assigned to a genus name. *Nitrobacter,* which was significantly more abundant at lower elevations, is a group that plays an important role in the nitrogen cycle by using energy from the oxidation of nitrate to fix CO_2_ *via* the Calvin cycle. *Nitrobacter* has been reported in glacier soils (Latha et al., 2009) and was proposed as the chemoautotrophic bacterium responsible for carbon fixation (Werner and Newton, 2005). Likewise, the other significant genera in our differential abundance analysis, *Cellulomonas* (Steven et al., 2006; Latha et al., 2009), *Oryzihumus* (Kwon et al., 2015; Zhang et al., 2016; Tolotti et al., 2020), and *Anaeromyxobacter* (Srinivas et al., 2011; Rime et al., 2015), were also previously reported in glacier microbiomes, but their ecological role has not yet been elucidated. *Anaeromyxobacter*, a common iron-reducing soil bacteria, has been shown to have the necessary molecular machinery for nitrogen fixation and assimilation of N_2_ gas by nitrogen (Masuda et al., 2017, 2020, 2021). The nitrogen-fixing capabilities of *Anaeromyxobacter* may play an essential role in the unique chemistry of soils at extreme altitudes in the Andes, which are characterized by low nitrogen content (Schmidt et al., 2008; Knelman et al., 2014; Hu et al., 2021). These three genera were significantly more abundant at lower elevations.

### 4.2. Human and animal-associated bacteria

Although mountain glaciers are extreme environments, and seldom visited by humans, they can be under different threats, including human activities. Human and animal fecal bacterial taxa have been reported in different glaciers, by detecting fecal microbial biomarkers (Zdanowski et al., 2017; Malešević et al., 2019). The Ruminococcaceae and Lachnospiraceae families, which were proposed as human and animal fecal biomarkers (Mclellan et al., 2013), have been found in all soil and glacier ice samples in the present study. The *Ruminococcus* genus was found in low-altitude glacier ice samples and represented 0.09% of the sequences in the *Firmicutes* phylum. *Ruminococcus* has been described as part of the bacterial consortia in sheep rumen (Krause et al., 1999). On the other hand, the genus *Faecalibacterium* considered a biomarker for poultry feces (Shen et al., 2013; Sun et al., 2016) was found in high-altitude glacier ice samples and represented 0.5% of the sequences in the *Firmicutes* phylum. The *Blautia* genus, found in both soil and glacier ice samples, with abundances ranging between 0.03and 0.05% respectively, has also been described as a biomarker for human feces (Koskey et al., 2014; Feng et al., 2018). The reasons for the presence of these fecal biomarkers are unknown but may be related to visitation by humans and native avian fauna, even though the samples were not collected on the touristic climbing routes. Nevertheless, the potential ecological significance of the detected biomarkers seems to be marginal due to their low relative abundance.

### 4.3. Glacier meta-community

Meta-community theory assumes that communities are not closed and isolated, but that they interact at various scales (Miller et al., 2018). One scale of interaction is spatial dynamics, which accounts for mass effect, rescue effect, colonization, dispersal, among other factors (Hanski and Gilpin, 1991). The ecology of glaciers can be classified as permanent habitats with indistinct boundaries (Leibold et al., 2004), since glacier soil and glacier ice are intimately in contact, allowing for colonization and dispersal effects (Wilhelm et al., 2013). Our findings support the concept of the meta-community in the CVC, as the intersections in the Venn diagram (Figure 4G) suggested that niches may occur through continuous ecosystems rather than having strictly categorical boundaries. This is particularly evident given that the central intersection of the Venn diagram held more than half of the detected families in this study (51.32%). The observed pattern in the meta-community at Cayambe, with a large overlap between communities, can be explained in terms of dispersal and colonization effects. Specifically, the hydraulic configuration of the glacial drainage may contribute to mass transport and the possibility of bacterial dispersal to colonize new glacier areas (Hotaling et al., 2017; Ortiz-Álvarez et al., 2020).

Our understanding of bacterial biodiversity and its drivers for mountain glaciers is mostly unquantified, overlooked, and underestimated due to the lack of data (Hotaling et al., 2017; Stibal et al., 2020). Therefore, this first assessment of the bacterial community in the CVC provides a new and useful perspective on the possible consequences of glacier retreat and climate change on microbial diversity and its associated ecosystems.

## Data availability statement

The datasets presented in this study can be found in online repositories. The names of the repository/repositories and accession number(s) can be found below: NCBI - PRJNA681925, SAMN16974609 until SAMN16974623.

## Author contributions

MD: sample collection, laboratory experiments, data analysis, and writing initial draft. PM-L: bioinformatic and data analysis. CQ-M: bioinformatic and data analysis. ER: sample collection and laboratory experiments. PC: sample collection. VA and WD: bioinformatic analysis. SA: writing – review and editing. FS: writing – review and editing. PJ-V: quantitative ecology analysis and writing – review and editing. CM: conceived the idea, sample collection, writing – review and editing, and grant administration. All authors contributed to the article and approved the submitted version.

## Funding

This work was supported by The World Academy of Sciences (TWAS), through the TWAS Research Grants Programme, under grant 16-172 RG/BIO/LA_I, and the Belgium Academy of Research and Higher Education (ARES – Académie de Recherche et d’Enseignement Supérieur) under project ARES-07-15K, through the ARES-UCE funding program. We extend our gratitude to both funding institutions.

## Conflict of interest

The authors declare that the research was conducted in the absence of any commercial or financial relationships that could be construed as a potential conflict of interest.

## Publisher’s note

All claims expressed in this article are solely those of the authors and do not necessarily represent those of their affiliated organizations, or those of the publisher, the editors and the reviewers. Any product that may be evaluated in this article, or claim that may be made by its manufacturer, is not guaranteed or endorsed by the publisher.

## Footnotes

^1^gitlab.com/ec.microbiome.proj/cayambe-microbiome-year-1

^2^https://figshare.com/ndownloader/files/40262224

^3^https://figshare.com/ndownloader/files/40262230

## Supplementary material

SUPPLEMENTARY FIGURE S1 Concentration or magnitude of physicochemical parameters between high- and low-altitude soil samples.

## References

[ref1] Anders S. Huber W. (2010). Differential expression analysis for sequence count data. Genome Biol. 11:R106. doi: 10.1186/gb-2010-11-10-r106, PMID: 20979621 PMC3218662

[ref2] Andrews S. (2010). FastQC. A Quality Control Tool for High Throughput Sequence Data. Available at: http://www.bioinformatics.babraham.ac.uk/projects/fastqc/(Accessed July 19, 2022).

[ref3] Baird R. Eaton A. Rice E. (2017). Standard Methods for the Examination of Water and Wastewater. 23rd Edn, Washington D.C: American Public Health Association.

[ref4] Bax V. Francesconi W. (2019). Conservation gaps and priorities in the tropical Andes biodiversity hotspot: implications for the expansion of protected areas. J. Environ. Manag. 232, 387–396. doi: 10.1016/j.jenvman.2018.11.086, PMID: 30500702

[ref5] Benjamini Y. Hochberg Y. (1995). Controlling the false discovery rate: a practical and powerful approach to multiple testing. J R Stat Soc Ser B 57, 289–300. doi: 10.2307/2346101

[ref6] Borda-Molina D. Montaña J. S. Zambrano M. M. Baena S. (2017). Mining lipolytic enzymes in community DNA from high Andean soils using a targeted approach. Anton. Leeuw. Int. J. Gen. Mol. Microbiol. 110, 1035–1051. doi: 10.1007/s10482-017-0877-8, PMID: 28523377

[ref7] Brown L. E. Milner A. M. Hannah D. M. (2007). Groundwater influence on alpine stream ecosystems. Freshw. Biol. 52, 878–890. doi: 10.1111/j.1365-2427.2007.01739.x

[ref8] Calvo P. Martinez C. Rico M. Rojas M. Oswald A. (2009). Microbiotic Biodiversity and their Functionality in Roots and Rhizosphere of Potato Plants. In 15th Triennial Symposium of the International Society for Tropical Root Crops (ISTRC) Proceedings (Lima); 110–116.

[ref9] Cauvy-Fraunié S. Dangles O. (2019). A global synthesis of biodiversity responses to glacier retreat. Nat Ecol Evol 3, 1675–1685. doi: 10.1038/s41559-019-1042-8, PMID: 31740846

[ref10] Cazzolla Gatti R. Dudko A. Lim A. Velichevskaya A. I. Lushchaeva I. V. Pivovarova A. V.. (2018). The last 50 years of climate-induced melting of the Maliy Aktru glacier (Altai Mountains, Russia) revealed in a primary ecological succession. Ecol. Evol. 8, 7401–7420. doi: 10.1002/ece3.4258, PMID: 30151159 PMC6106165

[ref11] Chan A. W. Y. Naphtali J. Schellhorn H. E. (2019). High-throughput DNA sequencing technologies for water and wastewater analysis. Sci. Prog. 102, 351–376. doi: 10.1177/0036850419881855 31818206 PMC10424514

[ref12] Cheng S. M. Foght J. M. (2007). Cultivation-independent and -dependent characterization of Bacteria resident beneath John Evans glacier. FEMS Microbiol. Ecol. 59, 318–330. doi: 10.1111/j.1574-6941.2006.00267.x, PMID: 17313581

[ref13] Ciccazzo S. Esposito A. Borruso L. Brusetti L. (2016). Microbial communities and primary succession in high altitude mountain environments. Ann. Microbiol. 66, 43–60. doi: 10.1007/s13213-015-1130-1

[ref14] Detienne M. Delmelle P. Guevara A. Samaniego P. Opfergelt S. Mothes P. A. (2017). Contrasting origin of two clay-rich debris flows at Cayambe volcanic complex, Ecuador. Bull. Volcanol. 79, 27–40. doi: 10.1007/s00445-017-1111-2

[ref15] Díaz M. Quiroz-Moreno C. Jarrín-V P. Piquer-Esteban S. Monfort-Lanzas P. Rivadeneira E.. (2022). Soil bacterial community along an altitudinal gradient in the Sumaco, a stratovolcano in the Amazon region. Front For Glob Change 5:738568. doi: 10.3389/ffgc.2022.738568

[ref16] Eisenhofer R. Minich J. J. Marotz C. Cooper A. Knight R. Weyrich L. S. (2019). Contamination in low microbial biomass microbiome studies: issues and recommendations. Trends Microbiol. 27, 105–117. doi: 10.1016/j.tim.2018.11.003, PMID: 30497919

[ref17] EPA. Laboratory Services and Applied Science Division. (2020a). Soil Sampling, Operating Procedure LSASDPROC-300-R4. Athens, Georgia.

[ref18] EPA. Laboratory Services and Applied Science Division. (2020b). Field Equipment Cleaning and Decontamination, Operating Procedure LSASDPROC-205-R4. Athens, Georgia.

[ref19] Feng S. Bootsma M. McLellan S. L. (2018). Human-associated Lachnospiraceae genetic markers improve detection of fecal pollution sources in urban waters. Appl. Environ. Microbiol. 84, 1–14. doi: 10.1128/AEM.00309-18, PMID: 29728386 PMC6029095

[ref20] Fierer N. McCain C. M. Meir P. Zimmermann M. Rapp J. M. Silman M. R.. (2011). Microbes do not follow the elevational diversity patterns of plants and animals. Ecology 92, 797–804. doi: 10.1890/10-1170.1 21661542

[ref21] Gallegos Castro E. Brito Chasiluisa C. Serrano Giné D. Galárraga Sánchez R. (2018). Análisis de la variación temporal y espacial de la cobertura glaciar del Nevado Cayambe, Ecuador, mediante fotografías aéreas e imágenes Landsat. GeoFocus Revista Internacional de Ciencia y Tecnología de la Información Geográfica 22, 97–113. doi: 10.21138/gf.577

[ref22] Gu Z. Eils R. Schlesner M. (2016). Complex heatmaps reveal patterns and correlations in multidimensional genomic data. Bioinformatics 32, 2847–2849. doi: 10.1093/bioinformatics/btw313, PMID: 27207943

[ref23] Guillier B. Chatelain J. L. (2006). Evidence for a seismic activity mainly constituted of hybrid events at Cayambe volcano, Ecuador. Interpretation in a iced-domes volcano context. Comptes Rendus Geosci 338, 499–506. doi: 10.1016/j.crte.2006.03.004

[ref24] Hanski I. Gilpin M. (1991). Metapopulation dynamics: brief history and conceptual domain. Biol. J. Linn. Soc. 42, 3–16. doi: 10.1111/j.1095-8312.1991.tb00548.x

[ref25] Hoham R. W. Remias D. (2020). Snow and glacial algae: a review. J. Phycol. 56, 264–282. doi: 10.1111/jpy.12952, PMID: 31825096 PMC7232433

[ref26] Hotaling S. Hood E. Hamilton T. L. (2017). Microbial ecology of mountain glacier ecosystems: biodiversity, ecological connections and implications of a warming climate. Environ. Microbiol. 19, 2935–2948. doi: 10.1111/1462-2920.13766, PMID: 28419666

[ref27] Hu W. Schmidt S. K. Sommers P. Darcy J. L. Porazinska D. L. (2021). Multiple-trophic patterns of primary succession following retreat of a high-elevation glacier. Ecosphere 12:e03400. doi: 10.1002/ecs2.3400

[ref28] Jacobsen D. Dangles O. (2012). Environmental harshness and global richness patterns in glacier-fed streams. Glob. Ecol. Biogeogr. 21, 647–656. doi: 10.1111/j.1466-8238.2011.00699.x

[ref29] Jost L. (2006). Entropy and diversity. Oikos 113, 363–375. doi: 10.1111/j.2006.0030-1299.14714.x

[ref30] Kandlikar G. S. Gold Z. J. Cowen M. C. Meyer R. S. Freise A. C. Kraft N. J. B.. (2018). Ranacapa: an R package and shiny web app to explore environmental DNA data with exploratory statistics and interactive visualizations. F1000Res 7:1734. doi: 10.12688/f1000research.16680.1, PMID: 30613396 PMC6305237

[ref31] Kim D. Hofstaedter C. E. Zhao C. Mattei L. Tanes C. Clarke E.. (2017). Optimizing methods and dodging pitfalls in microbiome research. Microbiome 5:52 (2017). doi: 10.1186/s40168-017-0267-5, PMID: 28476139 PMC5420141

[ref32] Klindworth A. Pruesse E. Schweer T. Peplies J. Quast C. Horn M.. (2013). Evaluation of general 16S ribosomal RNA gene PCR primers for classical and next-generation sequencing-based diversity studies. Nucleic Acids Res. 41, e1–e11. doi: 10.1093/nar/gks808, PMID: 22933715 PMC3592464

[ref33] Knelman J. E. Schmidt S. K. Lynch R. C. Darcy J. L. Castle S. C. Cleveland C. C.. (2014). Nutrient addition dramatically accelerates microbial community succession. PLoS One 9:e102609. doi: 10.1371/journal.pone.0102609, PMID: 25050551 PMC4106831

[ref34] Koskey A. M. Fisher J. C. Eren A. M. Ponce-Terashima R. Reis M. G. Blanton R. E.. (2014). Blautia and Prevotella sequences distinguish human and animal fecal pollution in Brazil surface waters. Environ. Microbiol. Rep. 6, 696–704. doi: 10.1111/1758-2229.12189, PMID: 25360571 PMC4247797

[ref35] Kozich J. J. Westcott S. L. Baxter N. T. Highlander S. K. Schloss P. D. (2013). Development of a dual-index sequencing strategy and curation pipeline for analyzing amplicon sequence data on the miseq illumina sequencing platform. Appl. Environ. Microbiol. 79, 5112–5120. doi: 10.1128/AEM.01043-13, PMID: 23793624 PMC3753973

[ref36] Krause D. O. Dalrymple B. P. Smith W. J. Mackie R. I. McSweeney C. S. (1999). 16S rDNA sequencing of Ruminococcus albus and *Ruminococcus flavefaciens*: design of a signature probe and its application in adult sheep. Microbiology (N Y) 145, 1797–1807. doi: 10.1099/13500872-145-7-1797, PMID: 10439419

[ref37] Kwon H. Y. Jung J. Y. Kim O. S. Laffly D. Lim H. S. Lee Y. K. (2015). Soil development and bacterial community shifts along the chronosequence of the Midtre Lovénbreen glacier foreland in Svalbard. J Ecol Environ 38, 461–476. doi: 10.5141/ecoenv.2015.049

[ref38] Lanzén A. Epelde L. Blanco F. Martín I. Artetxe U. Garbisu C. (2016). Multi-targeted metagenetic analysis of the influence of climate and environmental parameters on soil microbial communities along an elevational gradient. Sci. Rep. 6:28257. doi: 10.1038/srep28257, PMID: 27321429 PMC4913321

[ref39] Latha P. K. Soni R. Khan M. Marla S. S. Goel R. (2009). Exploration of Csp genes from temperate and glacier soils of the Indian Himalayas and in silico analysis of encoding proteins. Curr. Microbiol. 58, 343–348. doi: 10.1007/s00284-008-9344-0, PMID: 19159976

[ref40] Lee S. H. Jang I. Chae N. Choi T. Kang H. (2013). Organic layer serves as a hotspot of microbial activity and abundance in Arctic tundra soils. Microb. Ecol. 65, 405–414. doi: 10.1007/s00248-012-0125-8, PMID: 22983497

[ref41] Leibold M. A. Holyoak M. Mouquet N. Amarasekare P. Chase J. M. Hoopes M. F.. (2004). The metacommunity concept: a framework for multi-scale community ecology. Ecol. Lett. 7, 601–613. doi: 10.1111/j.1461-0248.2004.00608.x

[ref42] Liu Y. Yao T. Jiao N. Tian L. Hu A. Yu W.. (2011). Microbial diversity in the snow, a moraine lake and a stream in Himalayan glacier. Extremophiles 15, 411–421. doi: 10.1007/s00792-011-0372-5, PMID: 21468724

[ref43] Looby C. I. Maltz M. R. Treseder K. K. (2016). Below ground responses to elevation in a changing cloud forest. Ecol. Evol. 6, 1996–2009. doi: 10.1002/ece3.2025, PMID: 27066220 PMC4767876

[ref44] Love M. I. Huber W. Anders S. (2014). Moderated estimation of fold change and dispersion for RNA-seq data with DESeq2. Genome Biol. 15, 550–521. doi: 10.1186/s13059-014-0550-8, PMID: 25516281 PMC4302049

[ref45] Loveland-Curtze J. Miteva V. I. Brenchley J. E. (2009). *Herminiimonas glaciei* sp. nov., a novel ultramicrobacterium from 3042 m deep greenland glacial ice. Int. J. Syst. Evol. Microbiol. 59, 1272–1277. doi: 10.1099/ijs.0.001685-0 19502300

[ref46] Maechler M (2022). sfsmisc: Utilities from 'Seminar Fuer Statistik’ ETH Zurich. R Package Version 1.1-14. Available at: https://CRAN.R-project.org/package=sfsmisc (Accessed December 16, 2022).

[ref47] Małecki J. Lovell H. Ewertowski W. Górski Ł. Kurczaba T. Latos B.. (2018). The glacial landsystem of a tropical glacier: Charquini Sur, Bolivian Andes. Earth Surf. Process. Landf. 43, 2584–2602. doi: 10.1002/esp.4417

[ref48] Malešević M. Mirković N. Lozo J. Novović K. Filipić B. Kojić M.. (2019). Bacterial diversity among the sediments of glacial lakes in the western Balkans: exploring the impact of human population. Geomicrobiol J. 36, 261–270. doi: 10.1080/01490451.2018.1550128

[ref49] Marcon E. Hérault B. (2015). Entropart: an R package to measure and partition dive. J. Stat. Softw. 67, 1–26. doi: 10.18637/jss.v067.i08

[ref50] Margesin R. Shinner F. Marx J.-C. Gerday (2009). Psychrophiles from Biodiversity to Biotechnology. Berlin: Springer Science & Business Media.

[ref51] Masuda Y. Itoh H. Shiratori Y. Isobe K. Otsuka S. Senoo K. (2017). Predominant but previously-overlooked prokaryotic drivers of reductive nitrogen transformation in paddy soils, revealed by metatranscriptomics. Microbes Environ. 32, 180–183. doi: 10.1264/jsme2.ME16179 28442658 PMC5478542

[ref52] Masuda Y. Shiratori Y. Ohba H. Ishida T. Takano R. Satoh S.. (2021). Enhancement of the nitrogen-fixing activity of paddy soils owing to iron application. Soil Sci. Plant Nutr. 67, 243–247. doi: 10.1080/00380768.2021.1888629

[ref53] Masuda Y. Yamanaka H. Xu Z. X. Shiratori Y. Aono T. Amachi S.. (2020). Diazotrophic anaeromyxobacter isolates from soils. Appl. Environ. Microbiol. 86:e00956-20. doi: 10.1128/AEM.00956-20, PMID: 32532868 PMC7414960

[ref54] Mclellan S. L. Newton R. J. Vandewalle J. L. Shanks O. C. Huse S. M. Eren A. M.. (2013). Sewage reflects the distribution of human faecal Lachnospiraceae. Environ. Microbiol. 15, 2213–2227. doi: 10.1111/1462-2920.12092 23438335 PMC4043349

[ref55] McMurdie P. J. Holmes S. (2013). Phyloseq: an R package for reproducible interactive analysis and graphics of microbiome census data. PLoS One 8:e61217. doi: 10.1371/journal.pone.0061217, PMID: 23630581 PMC3632530

[ref56] Meier C. L. Rapp J. Bowers R. M. Silman M. Fierer N. (2010). Fungal growth on a common wood substrate across a tropical elevation gradient: temperature sensitivity, community composition, and potential for above-ground decomposition. Soil Biology Biochem 42, 1083–1090. doi: 10.1016/j.soilbio.2010.03.005

[ref57] Miller E. T. Svanbäck R. Bohannan B. J. M. (2018). Microbiomes as metacommunities: understanding host-associated microbes through metacommunity ecology. Trends Ecol. Evol. 33, 926–935. doi: 10.1016/j.tree.2018.09.002, PMID: 30266244

[ref58] Milner A. M. Brittain J. E. Castella E. Petts G. E. (2001). Trends of macroinvertebrate community structure in glacier-fed rivers in relation to environmental conditions: a synthesis. Freshw. Biol. 46, 1833–1847. doi: 10.1046/j.1365-2427.2001.00861.x

[ref59] Milner A. M. Brown L. E. Hannah D. M. (2009). Hydroecological response of river systems to shrinking glaciers. Hydrol. Process. 23, 62–77. doi: 10.1002/hyp.7197

[ref60] Monzier M. Samaniego P. Claude R. (1996). Le volcan Cayanbe (Équateur): son activité au cours des 5000 dernières années et les menaces qui en résultent. Bull. Inst. Fr. Etudes Andines 25, 389–397.

[ref61] Mukhia S. Kumar A. Kumari P. Kumar R. Kumar S. (2022). Multilocus sequence based identification and adaptational strategies of Pseudomonas sp. from the supraglacial site of Sikkim Himalaya. PLoS One 17:e0261178. doi: 10.1371/journal.pone.0261178, PMID: 35073328 PMC8786180

[ref62] Nayfach S. Roux S. Seshadri R. Udwary D. Varghese N. Schulz F.. (2020). A genomic catalog of Earth’s microbiomes. Nat. Biotechnol. 39, 499–509. doi: 10.1038/s41587-020-0718-6 33169036 PMC8041624

[ref63] Nottingham A. T. Fierer N. Turner B. L. Whitaker J. Ostle N. J. McNamara N. P.. (2018). Microbes follow Humboldt: temperature drives plant and soil microbial diversity patterns from the Amazon to the Andes. Ecology 99, 2455–2466. doi: 10.1002/ecy.2482, PMID: 30076592 PMC6850070

[ref64] Nottingham A. T. Whitaker J. Turner B. L. Salinas N. Zimmerman M. Malhi Y.. (2015). Climate warming and soil carbon in tropical forests: insights from an elevation gradient in the Peruvian Andes. Bioscience 65, 906–921. doi: 10.1093/biosci/biv109, PMID: 26955086 PMC4777015

[ref65] Obata H. Ishigaki H. Kawahara H. Yamade K. (1998). Purification and characterization of a novel cold-regulated protein from an ice-nucleating bacterium, *Pseudomonas fluorescens* KUIN-1. Biosci. Biotechnol. Biochem. 62, 2091–2097. doi: 10.1271/bbb.62.2091, PMID: 9972230

[ref66] Oerlemans J. (1994). Quantifying global warming from the retreat of glaciers. Science 264, 243–245. doi: 10.1126/science.264.5156.243, PMID: 17749022

[ref67] Oksanen J. Blanchet G. Friendly M. Kindt R. Legendre P. McGlinn D.. (2018). Vegan: Community Ecology Package. Available at: https://cran.r-project.org/web/packages/vegan/index.html (Accessed December 12, 2022).

[ref68] Ondov B. D. Bergman N. H. Phillippy A. M. (2011). Interactive metagenomic visualization in a web browser. BMC Bioinformatics 12:385. doi: 10.1186/1471-2105-12-385, PMID: 21961884 PMC3190407

[ref69] Ortiz-Álvarez R. Cáliz J. Camarero L. Casamayor E. O. (2020). Regional community assembly drivers and microbial environmental sources shaping bacterioplankton in an alpine lacustrine district (Pyrenees, Spain). Environ. Microbiol. 22, 297–309. doi: 10.1111/1462-2920.14848, PMID: 31680440

[ref70] Park H. J. Chae N. Sul W. J. Lee B. Y. Lee Y. K. Kim D. (2015). Temporal changes in soil bacterial diversity and humic substances degradation in subarctic tundra soil. Microb. Ecol. 69, 668–675. doi: 10.1007/s00248-014-0499-x, PMID: 25272964

[ref71] Peay K. von Sperber C. Cardarelli E. Toju H. Francis C. Chadwick O.. (2017). Convergence and contrast in the community structure of Bacteria, Fungi and Archaea along a tropical elevation-climate gradient. FEMS Microbiol. Ecol. 93, 1–12. doi: 10.1093/femsec/fix045, PMID: 28402397

[ref72] Peter H. Sommaruga R. (2016). Shifts in diversity and function of lake bacterial communities upon glacier retreat. ISME J. 10, 1545–1554. doi: 10.1038/ismej.2015.245, PMID: 26771929 PMC4852812

[ref73] R Core Team. (2020). R: A Language and Environment for Statistical Computing. R Foundation for Statistical Computing, Vienna, Austria.

[ref74] Rabatel A. Ceballos J. L. Micheletti N. Jordan E. Braitmeier M. González J.. (2018). Toward an imminent extinction of Colombian glaciers? Geogr. Ann. Ser. B 100, 75–95. doi: 10.1080/04353676.2017.1383015

[ref75] Rabatel A. Francou B. Soruco A. Gomez J. Cáceres B. Ceballos J. L.. (2013). Current state of glaciers in the tropical Andes: a multi-century perspective on glacier evolution and climate change. Cryosphere 7, 81–102. doi: 10.5194/tc-7-81-2013

[ref76] Ragot S. Zeyer J. Zehnder L. Reusser E. Brandl H. Lazzaro A. (2013). Bacterial community structures of an alpine apatite deposit. Geoderma 202–203, 30–37. doi: 10.1016/j.geoderma.2013.03.006

[ref77] Raper S. C. B. Braithwaite R. J. (2006). Low Sea level rise projections from mountain glaciers and icecaps under global warming. Nature 439, 311–313. doi: 10.1038/nature04448, PMID: 16421567

[ref78] Ren Z. Gao H. Elser J. J. (2017). Longitudinal variation of microbial communities in benthic biofilms and association with hydrological and physicochemical conditions in glacier-fed streams. Freshwater Sci 36, 479–490. doi: 10.1086/693133

[ref79] Rime T. Hartmann M. Brunner I. Widmer F. Zeyer J. Frey B. (2015). Vertical distribution of the soil microbiota along a successional gradient in a glacier forefield. Mol. Ecol. 24, 1091–1108. doi: 10.1111/mec.13051, PMID: 25533315

[ref80] Rognes T. Flouri T. Nichols B. Quince C. Mahé F. (2016). VSEARCH: a versatile open source tool for metagenomics. PeerJ 4, e2584–e2522. doi: 10.7717/peerj.2584, PMID: 27781170 PMC5075697

[ref81] Roslee A. F. A. Zakaria N. N. Convey P. Zulkharnain A. Lee G. L. Y. Gomez-Fuentes C.. (2020). Statistical optimisation of growth conditions and diesel degradation by the Antarctic bacterium, *Rhodococcus* sp. strain AQ5–07. Extremophiles 24, 277–291. doi: 10.1007/s00792-019-01153-0, PMID: 31863235

[ref82] Samaniego P. Monzier M. Robin C. Hall M. L. (1998). Late Holocene eruptive activity at Nevado Cayambe volcano, Ecuador. Bull. Volcanol. 59, 451–459. doi: 10.1007/s004450050203

[ref83] Schloss P. D. (2020). Reintroducing mothur: 10 years later. Appl. Environ. Microbiol. 86, 1–13. doi: 10.1128/AEM.02343-19 PMC6952234 31704678

[ref84] Schloss P. D. Westcott S. L. Ryabin T. Hall J. R. Hartmann M. Hollister E. B.. (2009). Introducing mothur: open-source, platform-independent, community-supported software for describing and comparing microbial communities. Appl. Environ. Microbiol. 75, 7537–7541. doi: 10.1128/AEM.01541-09, PMID: 19801464 PMC2786419

[ref85] Schmidt S. K. Nemergut D. R. Miller A. E. Freeman K. R. King A. J. Seimon A. (2009). Microbial activity and diversity during extreme freeze–thaw cycles in periglacial soils, 5400 m elevation, cordillera Vilcanota, Perú. Extremophiles 13, 807–816. doi: 10.1007/s00792-009-0268-9, PMID: 19597697

[ref86] Schmidt S. K. Reed S. C. Nemergut D. R. Stuart Grandy A. Cleveland C. C. Weintraub M. N.. (2008). The earliest stages of ecosystem succession in high-elevation (5000 metres above sea level), recently deglaciated soils. Proc. Biol. Sci. 275, 2793–2802. doi: 10.1098/rspb.2008.0808, PMID: 18755677 PMC2605835

[ref87] Schütte U. M. E. Abdo Z. Foster J. Ravel J. Bunge J. Solheim B.. (2010). Bacterial diversity in a glacier foreland of the high Arctic. Mol. Ecol. 19, 54–66. doi: 10.1111/j.1365-294X.2009.04479.x, PMID: 20331770

[ref88] Seok Y. J. Song E. J. Cha I. T. Lee H. Roh S. W. Jung J. Y.. (2016). Microbial community of the Arctic soil from the glacier foreland of Midtre Lovénbreen in Svalbard by metagenome analysis. Microbiol Biotechnol Lett 44, 171–179. doi: 10.4014/mbl.1601.01003

[ref89] Shen Z. Duan C. Zhang C. Carson A. Xu D. Zheng G. (2013). Using an intervening sequence of Faecalibacterium 16S rDNA to identify poultry feces. Water Res. 47, 6415–6422. doi: 10.1016/j.watres.2013.08.013, PMID: 24011842

[ref90] Shen C. Gunina A. Luo Y. Wang J. He J.-Z. Kuzyakov Y.. (2020). Contrasting patterns and drivers of soil bacterial and fungal diversity across a mountain gradient. Environ. Microbiol. 22, 3287–3301. doi: 10.1111/1462-2920.15090, PMID: 32436332

[ref91] Shi Y. Liu S. (2000). Estimation on the response of glaciers in China to the global warming in the 21st century. Chin. Sci. Bull. 45, 668–672. doi: 10.1007/BF02886048

[ref92] Simon C. Wiezer A. Strittmatter A. W. Daniel R. (2009). Phylogenetic diversity and metabolic potential revealed in a glacier ice metagenome. Appl. Environ. Microbiol. 75, 7519–7526. doi: 10.1128/AEM.00946-09, PMID: 19801459 PMC2786415

[ref93] Singh D. Lee-Cruz L. Kim W.-S. Kerfahi D. Chun J. Adams J. (2014). Strong elevational trends in soil bacterial community composition on Mt. Halla. South Korea. Soil Biol. Biochem. 68, 140–149. doi: 10.1016/j.soilbio.2013.09.027

[ref94] Sorg A. Kääb A. Roesch A. Bigler C. Stoffel M. (2015). Contrasting responses of central Asian rock glaciers to global warming. Sci. Rep. 5, 1–6. doi: 10.1038/srep08228, PMID: 25657095 PMC4319170

[ref95] Srinivas T. N. R. Singh S. M. Pradhan S. Pratibha M. S. Kishore K. H. Singh A. K.. (2011). Comparison of bacterial diversity in proglacial soil from Kafni glacier, Himalayan Mountain ranges, India, with the bacterial diversity of other glaciers in the world. Extremophiles 15, 673–690. doi: 10.1007/s00792-011-0398-8, PMID: 21918795

[ref96] Staley J. T. (1997). Biodiversity: are microbial species threatened?: commentary. Curr. Opin. Biotechnol. 8, 340–345. doi: 10.1016/S0958-1669(97)80014-6 9206017

[ref97] Steven B. Léveillé R. Pollard W. H. Whyte L. G. (2006). Microbial ecology and biodiversity in permafrost. Extremophiles 10, 259–267. doi: 10.1007/s00792-006-0506-3 16550305

[ref98] Stibal M. Bradley J. A. Edwards A. Hotaling S. Zawierucha K. Rosvold J.. (2020). Glacial ecosystems are essential to understanding biodiversity responses to glacier retreat. Nat Ecol Evol 4, 686–687. doi: 10.1038/s41559-020-1163-0, PMID: 32231325

[ref99] Stolz J. F. (2017). Gaia and her microbiome. FEMS Microbiol. Ecol. 93, 1–13. doi: 10.1093/femsec/fiw247 27940647

[ref100] Sun D. Duan C. Shang Y. Ma Y. Tan L. Zhai J.. (2016). Application of Faecalibacterium 16S rDNA genetic marker for accurate identification of duck faeces. Environ. Sci. Pollut. Res. 23, 7639–7647. doi: 10.1007/s11356-015-6024-z, PMID: 26743644

[ref101] Talukdar M. Bora T. C. Jha D. K. (2016). “Micromonospora: a potential source of antibiotic BT” in Bioprospecting of Indigenous Bioresources of North-East India. ed. Purkayastha J. (Singapore: Springer Singapore), 195–213.

[ref102] Tan B. F. Ng C. Nshimyimana J. P. Loh L. L. Gin K. Y. H. Thompson J. R. (2015). Next-generation sequencing (NGS) for assessment of microbial water quality: current progress, challenges, and future opportunities. Front. Microbiol. 6:1027. doi: 10.3389/fmicb.2015.01027, PMID: 26441948 PMC4585245

[ref103] Tolotti M. Cerasino L. Donati C. Pindo M. Rogora M. Seppi R.. (2020). Alpine headwaters emerging from glaciers and rock glaciers host different bacterial communities: ecological implications for the future. Sci. Total Environ. 717:137101. doi: 10.1016/j.scitotenv.2020.137101, PMID: 32065887

[ref104] Vásquez-Ponce F. Higuera-Llantén S. Pavlov M. S. Marshall S. H. Olivares-Pacheco J. (2018). Phylogenetic MLSA and phenotypic analysis identification of three probable novel Pseudomonas species isolated on King George Island, south Shetland, Antarctica. Brazilian J Microbiol 49, 695–702. doi: 10.1016/j.bjm.2018.02.005, PMID: 29598976 PMC6175711

[ref105] Venables W. N. Ripley B. D. (2002). Modern Applied Statistics with S. New York: Springer.

[ref106] Wang Q. Garrity G. M. Tiedje J. M. Cole J. R. (2007). Naive Bayesian classifier for rapid assignment of rRNA sequences into the new bacterial taxonomy. Appl. Environ. Microbiol. 73, 5261–5267. doi: 10.1128/AEM.00062-07, PMID: 17586664 PMC1950982

[ref107] Werner D. Newton W. (2005). Nitrogen Fixation in Agriculture, Forestry, Ecology, and the Environment. Dordrecht, Netherlands: Springer

[ref108] Westcott S. L. Schloss P. D. (2017). OptiClust, an improved method for assigning amplicon-based sequence data to operational taxonomic units. mSphere 2, 1–11. doi: 10.1128/mspheredirect.00073-17 PMC5343174 28289728

[ref109] Wilhelm L. Besemer K. Fasching C. Urich T. Singer G. A. Quince C.. (2014). Rare but active taxa contribute to community dynamics of benthic biofilms in glacier-fed streams. Environ. Microbiol. 16, 2514–2524. doi: 10.1111/1462-2920.12392, PMID: 24428193

[ref110] Wilhelm L. Singer G. A. Fasching C. Battin T. J. Besemer K. (2013). Microbial biodiversity in glacier-fed streams. ISME J. 7, 1651–1660. doi: 10.1038/ismej.2013.44, PMID: 23486246 PMC3721114

[ref111] Wu Y. P. Zhang Y. Bi Y. M. Sun Z. J. (2015). Biodiversity in saline and non-saline soils along the Bohai Sea coast, China. Pedosphere 25, 307–315. doi: 10.1016/S1002-0160(15)60015-7

[ref112] Xiang S. R. Shang T. C. Chen Y. Jing Z. F. Yao T. (2009). Dominant bacteria and biomass in the Kuytun 51 glacier. Appl. Environ. Microbiol. 75, 7287–7290. doi: 10.1128/AEM.00915-09, PMID: 19749065 PMC2786530

[ref113] Zdanowski M. K. Bogdanowicz A. Gawor J. Gromadka R. Wolicka D. Grzesiak J. (2017). Enrichment of cryoconite hole anaerobes: implications for the subglacial microbiome. Microb. Ecol. 73, 532–538. doi: 10.1007/s00248-016-0886-6, PMID: 27822618 PMC5348551

[ref114] Zhang B. Wu X. Zhang W. Chen X. Zhang G. Ai X.. (2016). Diversity and succession of Actinobacteria in the forelands of the Tianshan glacier, China. Geomicrobiol J. 33, 716–723. doi: 10.1080/01490451.2015.1085468

